# Clustering of Resting State Networks

**DOI:** 10.1371/journal.pone.0040370

**Published:** 2012-07-09

**Authors:** Megan H. Lee, Carl D. Hacker, Abraham Z. Snyder, Maurizio Corbetta, Dongyang Zhang, Eric C. Leuthardt, Joshua S. Shimony

**Affiliations:** Washington University School of Medicine, Saint Louis, Missouri, United States of America; Banner Alzheimer’s Institute, United States of America

## Abstract

**Background:**

The goal of the study was to demonstrate a hierarchical structure of resting state activity in the healthy brain using a data-driven clustering algorithm.

**Methodology/Principal Findings:**

The fuzzy-c-means clustering algorithm was applied to resting state fMRI data in cortical and subcortical gray matter from two groups acquired separately, one of 17 healthy individuals and the second of 21 healthy individuals. Different numbers of clusters and different starting conditions were used. A cluster dispersion measure determined the optimal numbers of clusters. An inner product metric provided a measure of similarity between different clusters. The two cluster result found the task-negative and task-positive systems. The cluster dispersion measure was minimized with seven and eleven clusters. Each of the clusters in the seven and eleven cluster result was associated with either the task-negative or task-positive system. Applying the algorithm to find seven clusters recovered previously described resting state networks, including the default mode network, frontoparietal control network, ventral and dorsal attention networks, somatomotor, visual, and language networks. The language and ventral attention networks had significant subcortical involvement. This parcellation was consistently found in a large majority of algorithm runs under different conditions and was robust to different methods of initialization.

**Conclusions/Significance:**

The clustering of resting state activity using different optimal numbers of clusters identified resting state networks comparable to previously obtained results. This work reinforces the observation that resting state networks are hierarchically organized.

## Introduction

The work of Biswal and colleagues [1] provided the first demonstration of synchrony between right and left sensorimotor areas. Since then, intrinsic activity has been studied and other resting state networks (RSNs) have been described, including the default mode network (DMN) [2], [3], a collection of spatially distinct regions that are active at rest and deactivated by tasks. Various other RSNs, or regions of the brain disparate in space but synchronous in time, have been described [4]–[6]. These include the visual network (VIS) [4], [5], the ventral attention network (VAN) [7], [8], and the frontoparietal control (FPC) network [9]–[11]. Many of these RSNs have been confirmed using a variety of methods [4], [5], [7], [8], [10]–[13].

A large scale view of the organization of resting state activity was offered by Fox et al. [14], who described two anticorrelated systems in the brain. This finding was supported by a k-means clustering analysis by Golland et al. [15], who also demonstrated two large systems, one primarily involving the DMN, and the other centered on the somatomotor network (SMN).

Although some of these RSNs are well defined, further work is needed to determine the relationships of these networks to each other and to the brain as a whole. Subcortical structures also need to be explored in this context. In a recent study using ICA followed by hierarchical clustering, Doucet et al. [12] found two competing systems. Three modules comprised the task-negative system and two modules comprised the task-positive system. Using a graph theoretic approach, Power et al. [10] found the task negative system to be comprised of the DMN, whereas the task positive system was comprised of the DAN, the FPC network, and the cingulo-opercular network.

In addition to insights on the physiology of the normal brain, a better understanding of the relationships between RSNs and the larger scale organization of the brain could contribute to increased knowledge about various neurological and psychiatric diseases. Resting state fMRI has already provided insights on several diseases [16], [17]. The DMN has been implicated in many studies, showing disruptions in functional connectivity in patients with Alzheimer’s disease [18], [19], schizophrenia [20], and depression [21]. However, further work is needed to explore other possible resting state biomarkers of these diseases. An effective and objective method for analyzing resting state data will also facilitate the use of this information in the clinical setting.

In the present study, we apply a clustering algorithm to explore the hierarchical organization of resting state activity in cortical and subcortical gray matter. The fuzzy-c-means algorithm [22] is an extension of the k-means algorithm that allows for a weighted classification of each voxel to each cluster, rather than all or none membership. This data driven algorithm has the advantage of requiring little *a priori* information. Using a cluster dispersion measure, we find optimal numbers of clusters and relate these results to the two large scale anticorrelated systems.

## Results

### Two Clusters

**Figure 1:**
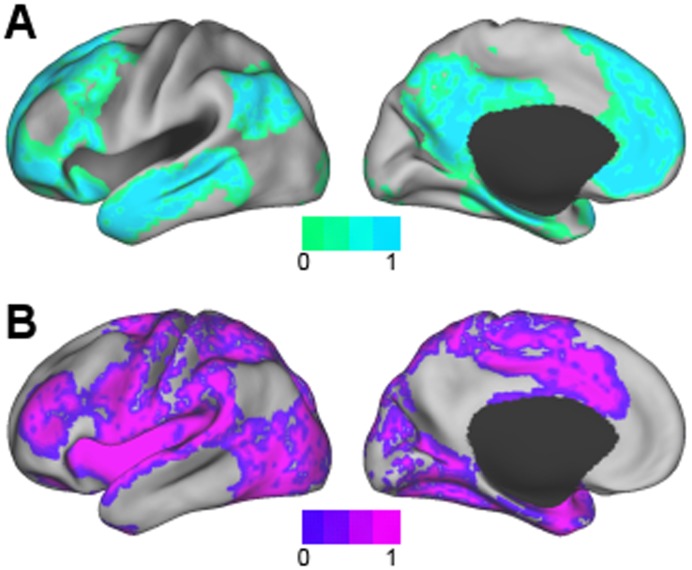
The two cluster result found the A) task-negative and B) task-positive systems.

The two cluster case is of interest in the context of prior studies that identified task-negative and task-positive systems [12], [14], [15]. The result of clustering the data with two clusters is shown in Figure 1. The first cluster included the DMN, as well as areas of the FPC and LAN networks, including the caudate. In contrast, the second cluster included somatomotor, visual areas, and dorsal attention areas. The centroids (Equation 2) of these two clusters were temporally anti-correlated with an inner product of −0.93.

### Optimal Numbers of Clusters

**Figure 2:**
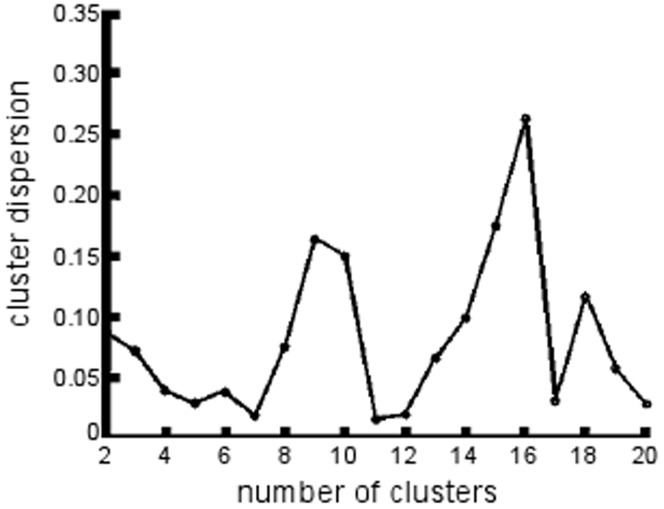
The cluster dispersion metric (**Equation 4**) for different numbers of clusters. Cluster dispersion was minimized for seven, eleven, and seventeen clusters.

To determine the optimal numbers of clusters, the clustering algorithm was run for 2–20 clusters. Based on the cluster dispersion metric (Equation 4), 7, 11, and 17 clusters were local minima (Figure 2).

### Seven Clusters

**Figure 3:**
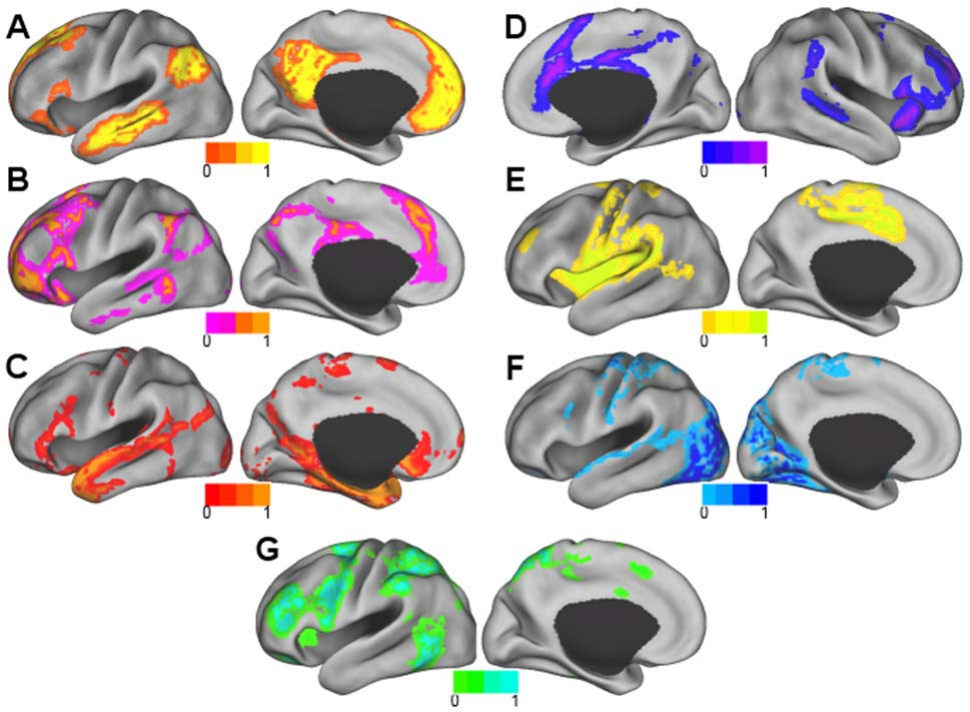
The seven cluster result. A) DMN, B) FPC network, C) LAN network, D) VAN, E) SMN, F) VIS network, and G) DAN. The right hemisphere is displayed for the VAN because it was right lateralized.

**Figure 4:**
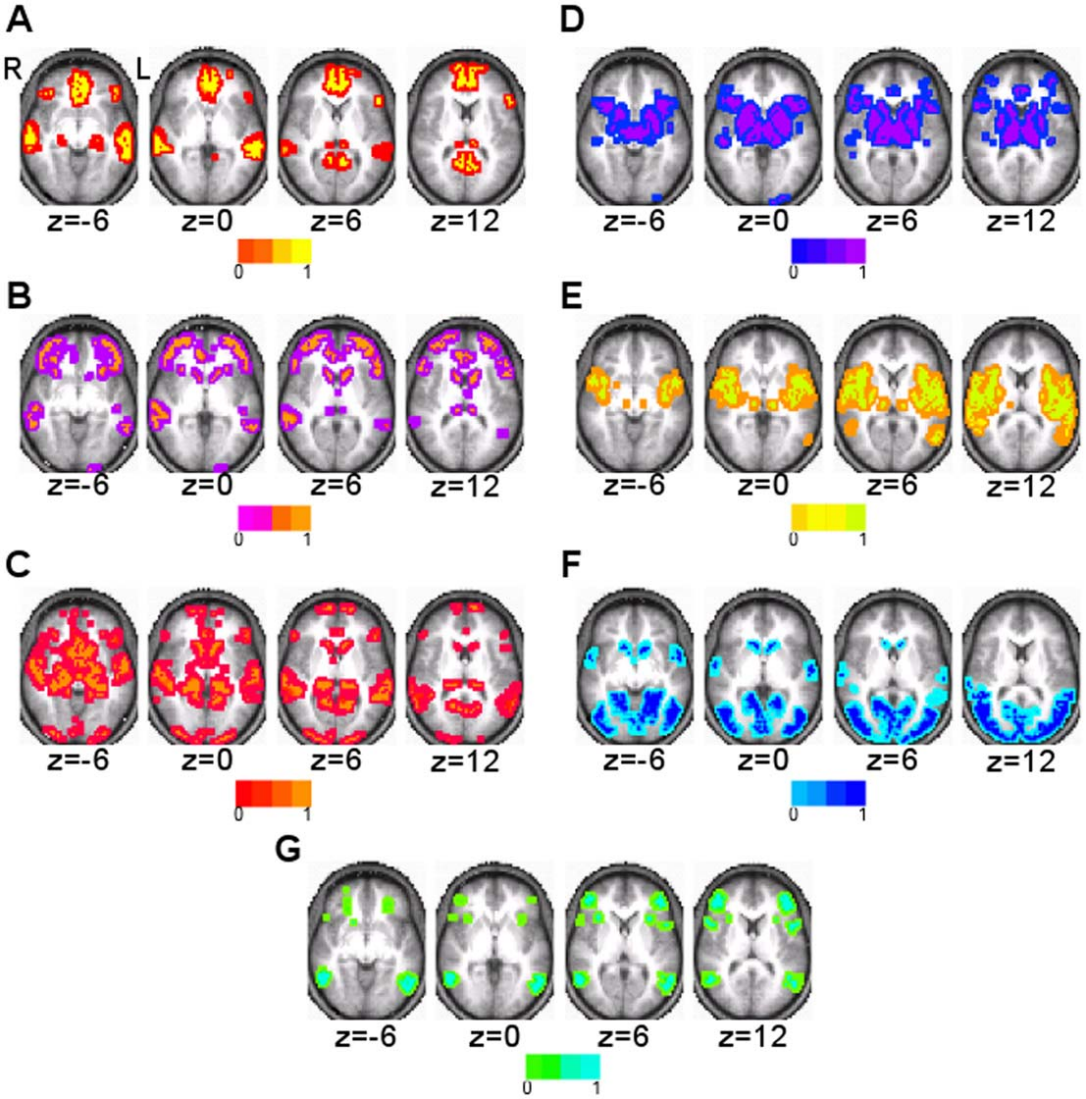
Subcortical involvement of the seven cluster result. Axial slices are shown in radiologic convention. A) DMN, B) FPC network, C) LAN network, D) VAN, E) SMN, F) VIS network, and G) DAN.

Surface maps of the seven clusters that were found using the clustering algorithm and random initialization are shown in Figure 3. Axial slices at the levels of subcortical gray matter are shown in Figure 4. They were identifiable as DMN, FPC, LAN, VAN, SMN, VIS, and DAN based on visual similarity with past studies. The FPC network also included the caudate (Figure 4B). In addition to Broca’s and Wernicke’s areas, the LAN network involved inferior temporal areas and bilateral caudate (Figure 3C and Figure 4C). The VAN involved of the basal ganglia and thalamus (Figure 4D). In addition to the pre- and post-central gyri and the supplementary motor area (SMA), the SMN also included the insular cortex and areas of the thalamus, including the ventral lateral posterior nuclei and anterior pulvinar nuclei (Figure 3E and Figure 4E). The VIS network also involved the caudate (Figure 4F).

**Figure 5:**
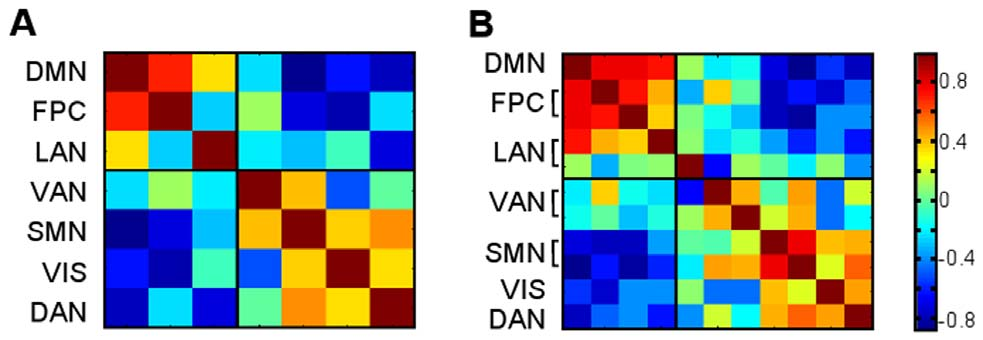
Temporal inner products (**Equation 6**) between centroids from the A) seven cluster result and B) eleven cluster result.

The temporal similarity measures (Equation 6) between centroids obtained by the seven cluster result are shown in Figure 5A. The DMN was positively correlated with the FPC network (0.70) and the LAN network (0.37). The DMN was most anticorrelated with the SMN (−0.82) but also anticorrelated with the VAN (−0.20), the VIS network (−0.60), and the DAN (−0.74).

**Figure 6:**
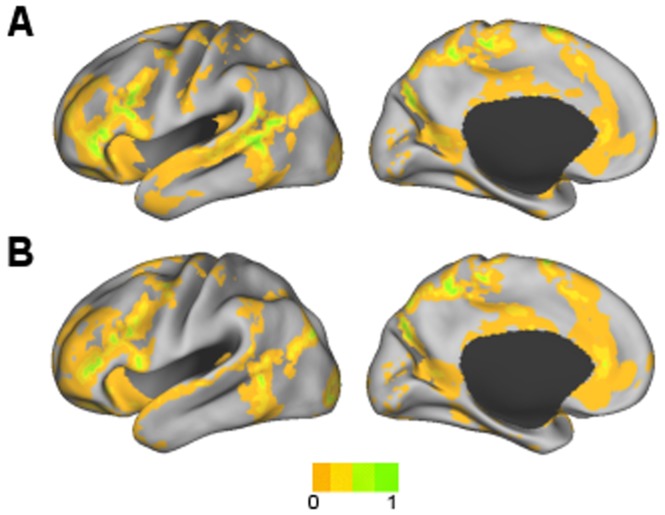
The degree of uncertainty (**Equation 7**) as shown by the geometric mean of weights for each voxel in the A) seven cluster result and B) eleven cluster result.

Figure 6A shows the voxelwise degree of uncertainty (Equation 7) for the seven cluster result. Higher values indicate greater shared membership. When compared to the seven cluster result using the spatial similarity measure, the networks most related to the uncertainty map were the LAN network (0.17), the FPC network (0.12), and the VAN (0.10).

The data were also clustered into seven clusters using non-random initialization (see Methods). The resulting seven weight vectors were identical to those found with random initialization, yielding spatial similarity measures of 1.0 in all cases. 100 runs of the algorithm using random initialization also yielded consistent results. For each of the seven clusters, the spatial similarity measure was 1.0 for 95 of 100 runs. The percentages of runs that had a spatial similarity measure of greater than 0.90 were: 100% for DMN, 99% for FPC, 100% for LAN, 95% for VAN, 98% for SMN, 95% for VIS, and 95% for DAN.

The average data of the group of 21 subjects were also clustered into seven clusters using random initialization. The spatial and temporal similarity measures between this group and the group of 17 subjects are shown in Table 1.

**Table 1:** Similarity measures for clustering results of the second study group compared to the first.

#clusters	Similarity	DMN	FPC		LAN		VAN		SMN		VIS	DAN
Seven	Spatial	0.92	0.78		0.83		0.84		0.87		0.84	0.83
	Temporal	1.00	0.96		0.96		0.96		0.99		0.97	0.99
Eleven	Spatial	0.80	0.62	0.39	0.81	0.85	0.42	0.94	0.88	0.90	0.89	0.76
	Temporal	0.99	0.95	0.49	0.94	0.97	0.39	1.00	0.99	1.00	0.99	0.97

### Eleven and 17 Cluster Results

**Figure 7:**
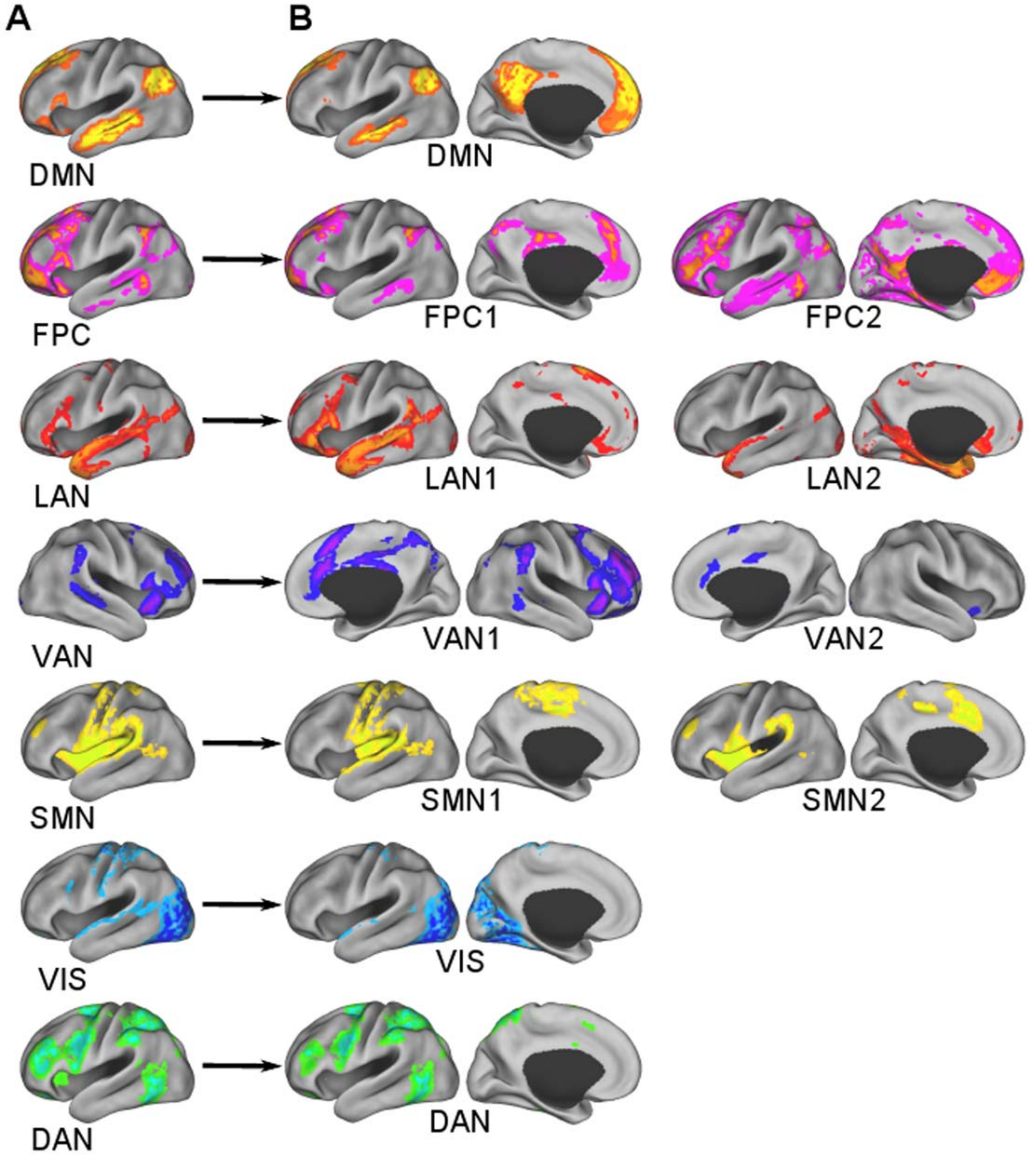
Some RSNs from the A) seven cluster result were subdivided in the B) eleven cluster result. The right hemisphere is displayed for the VAN and its subdivisions because they were right lateralized.

**Figure 8:**
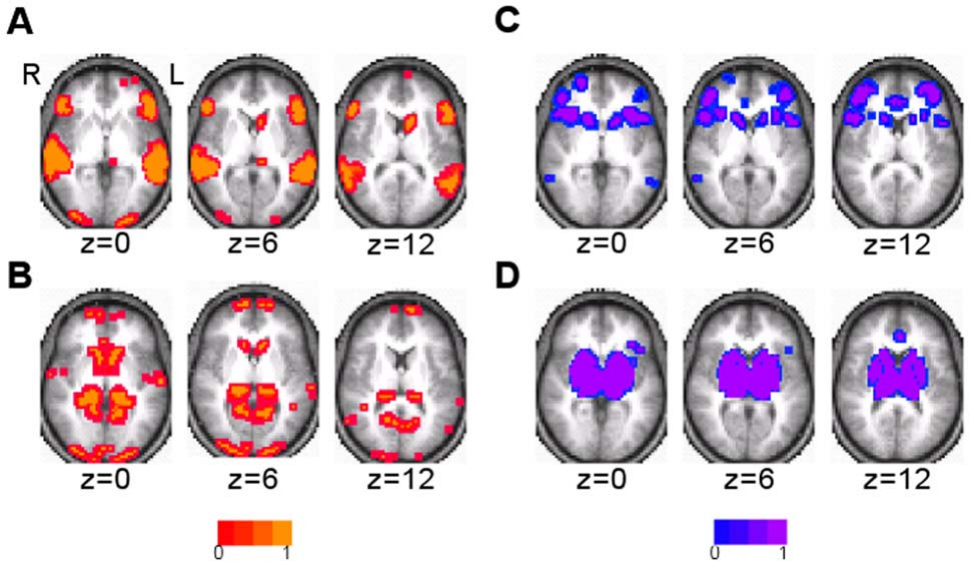
In the eleven cluster solution, the LAN network and VAN were divided into approximately cortical and subcortical clusters. Axial slices are shown in radiological convention of A) LAN1, B) LAN2, C) VAN1, D) VAN2.

The eleven cluster result subdivided some of the seven RSNs (Figure 7). Split RSNs were named using the RSN acronym with a 1 or 2 added. DMN, VIS, and DAN were preserved with spatial similarity measures of 0.93, 0.96, and 0.90, respectively. Each of the remaining four RSNs was subdivided into two clusters. The two divisions of the FPC network (FPC1 and FPC2) are shown in Figure 7B. The LAN network was also divided into two components (LAN1 and LAN2), with one consisting primarily of Broca’s and Wernicke’s areas (Figure 8A) and the other component including the caudate and regions of the inferior temporal lobe (Figure 8B). The VAN was also divided into two components (VAN1 and VAN2), the first containing mainly cortical areas (Figure 8C), and the second predominated by the basal ganglia and thalamus (Figure 8D). The first division of the SMN (SMN1) included the pre- and post-central gyri, SMA, posterior insular cortex, and part of the superior temporal gyrus. The second division of the SMN (SMN2) included anterior insular cortex and the pre-SMA (Figure 7B).

The average data of the group of 21 subjects were also clustered into eleven clusters using random initialization. The spatial (Equation 5) and temporal similarity measures (Equation 6) between this group and the group of 17 subjects are shown in Table 1. The spatial similarity between the results of the first and second group was the least for FPC2.

The temporal similarities (Equation 6) between the centroids of the eleven cluster result are shown in Figure 5B. The DMN was again positively correlated with the two divisions of the FPC network (0.79 and 0.82). LAN1 was more correlated with the DMN (0.72) compared to LAN2 (0.094). The VIS network, the DAN, and both divisions of the SMN were anticorrelated to the DMN. The two divisions of the VAN were most weakly anticorrelated with the DMN (−0.24 and −0.15).

Figure 6B shows the degree of uncertainty (Equation 7) for the eleven cluster result. When compared to the eleven cluster result using the spatial similarity measure, the uncertainty map was most similar to FPC2 (0.39). It was also similar to VAN1 (0.29), and less similar to LAN1 (0.08), LAN2 (0.06), and FPC1 (0.01).

The seventeen cluster result subdivided the RSNs further (Figure S1). As compared to the 7 cluster result the DMN, VIS network, DAN, and VAN were split into two components, and the FPC network, LAN network, and SMN, were split into three. Notably, the VIS network split into foveal and peripheral divisions. However, compared to the eleven cluster result, the subdivisions in the seventeen cluster result were less spatially distinct from each other, especially the first two subdivisions of FPC and LAN networks and the second two subdivisions of SMN.

### Comparison of the Two Cluster Result with the Seven and Eleven Cluster Results

**Figure 9:**
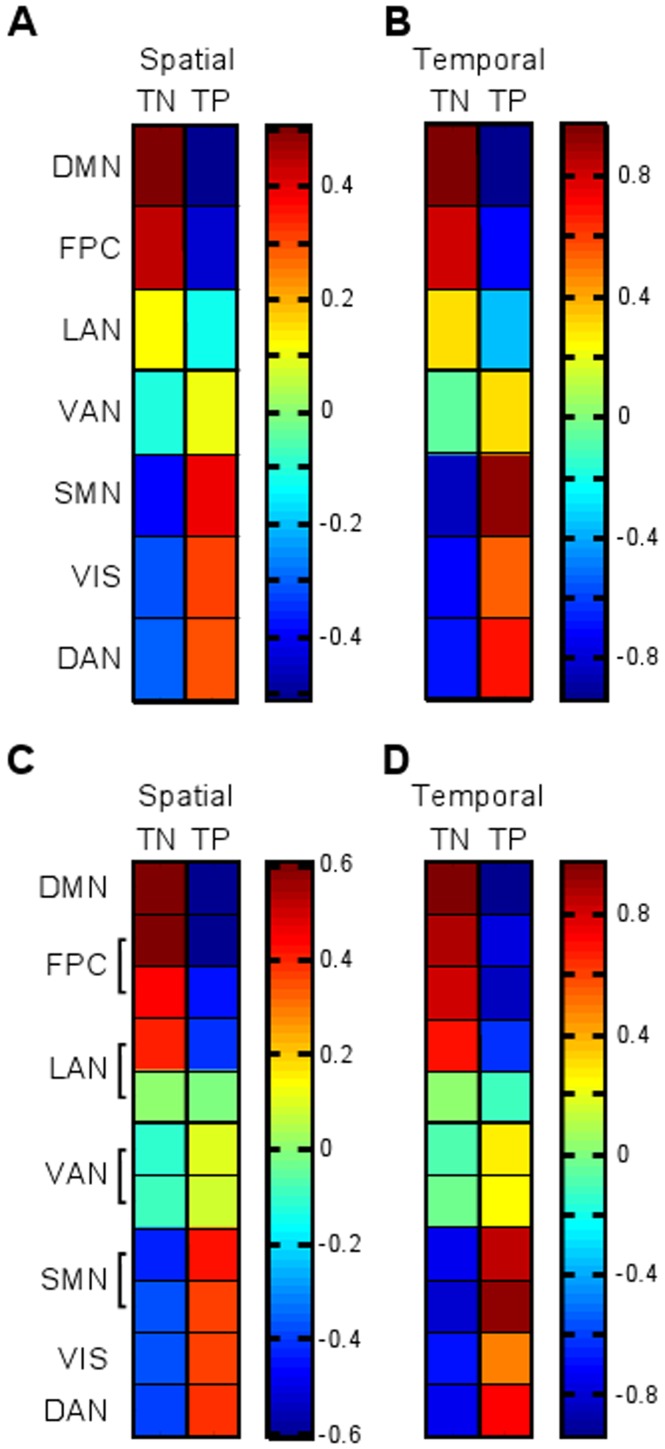
The relationships between the seven and eleven cluster results and the task-negative (TN) and task-positive (TP) systems from the two cluster result. The A) spatial (Equation 5) and B) temporal (Equation 6) inner products between the two cluster and seven cluster results. The C) spatial (Equation 5) and D) temporal (Equation 6) inner products between the two cluster and eleven cluster results.

To demonstrate a hierarchical relationship between the different numbers of clusters, the two cluster result was compared to the seven and eleven cluster results. When the seven clusters were compared with the two cluster result using the spatial similarity measure (Equation 5) (Figure 9A), the DMN, FPC and LAN networks had positive inner products with the task-negative system and negative inner products with the extrinsic system. The VAN, SMN, VIS network, and DAN had positive inner products with the task-positive system and negative inner products with the task-negative system. The clusters with the smallest inner product magnitudes were the LAN network (0.14 with the task-negative system, −0.14 with the task-positive system) and the VAN (0.12 with the task-positive system, −0.12 with the task-negative system).

The same pattern of positive and negative inner products was seen when the temporal similarity measure (Equation 6) was used to compare the two and seven cluster centroids (Figure 9B). The LAN network and VAN again had the smallest magnitudes. The LAN network centroid was temporally correlated with the task-negative system (0.29) and anticorrelated with the task-positive system (−0.34). The VAN centroid was temporally correlated with the task-positive system (0.30) and weakly anticorrelated with the task-negative system (−0.051). This separation is also emphasized in Figure 5 by the thick black separating lines.

The eleven cluster result was also compared with the two cluster result (Figure 9C and Figure 9D). In both the spatial (Equation 5) and temporal (Equation 6) comparisons, the DMN, FPC1 and FPC2, and LAN1 and LAN2 were positively correlated with the task-negative system and negatively correlated with the task-positive system. VAN1 and VAN2, SMN1 and SMN2, VIS, and DAN were positively correlated with the task-positive system and negatively correlated with the task-negative system. Among the task-negative components, LAN2 had the smallest positive inner product with the task-negative system (0.0035 spatially and 0.042 temporally). In contrast, LAN1 had higher inner products with the task-negative system (0.35 spatially and 0.70 temporally). Among the task-positive clusters, VAN1 and VAN2 had the smallest positive inner products with the task-positive system (VAN1 0.082 spatially and 0.26 temporally, VAN2 0.080 spatially and 0.24 temporally).

### Relationships between Cluster Results between Two and Seven

An additional feature of our analysis is the availability of data on the transition between the two and seven cluster results. Accordingly, details of the transition between two and seven, accompanied by the improvement in the cluster dispersion measure (Figure 2) can identify features of the hierarchical transition. We provide this information for the interested reader in Tables S1 and S2.

## Discussion

### Hierarchical Organization of RSNs

We demonstrated a hierarchical organization of RSNs by identifying optimal numbers of clusters and relating the seven and eleven cluster result to the two cluster result. Our two cluster result represents a large scale division of resting state activity that also accommodates the finer divisions at the levels of seven and eleven clusters. The two cluster result identified the task-negative system, which in past studies has been shown to include the DMN, and the task-positive system, which has been shown to include the SMN and the DAN [12], [14], [15]. By spatially and temporally comparing the seven cluster result to the two cluster result, we demonstrated that the DMN, FPC network, and LAN network are components of the task-negative system, whereas the VAN, SMN, VIS network, and DAN are components of the task-positive system. These relationships also held when comparing the eleven cluster result with the task-negative and task-positive systems.

We also showed that this pattern exists among the seven and eleven cluster centroids. As an example, the DMN centroid was positively correlated to those of the FPC and LAN networks but negatively correlated to those of the VAN, SMN, VIS network, and DAN.

The hierarchical organization found by our method differed in some respects from that found by Doucet et al. [12]. For example, we found four components of the task-positive system and found the VAN to be more closely related with the task-positive system. However, Doucet et al. did find that the module containing “salience” areas was positively correlated with their somatosensory-attentional module from the task-positive system [12].

Our inclusion of the FPC network in the task-negative system also contrasted with Power et al. [10] who found it to be part of the task-positive system, along with the DAN and the cingulo-opercular control network. Notably, the cingulo-opercular network in [10] is contained within our larger VAN network. In our analysis, the centroids of the FPC clusters in both the seven and eleven cluster results were highly correlated with the DMN centroids. The positive relationship between the FPC network and the DMN is supported by task based fMRI studies which found that autobiographical planning as opposed to visuospatial planning [23] or rest or movie watching as opposed to finger tapping [24] were associated with positive correlations between the DMN and the FPC network.

The ability to assign a voxel to more than one network illuminates other findings of interest, such as the distribution of the DAN, FPC, and the DMN in the superior and inferior parietal lobule [11], and the importance of the anterior insula with involvement of the three control networks, VAN, FPC, and DAN [10], [13]. The area of the anterior insula has been shown to be a common area of activation in many fMRI experiments [25]. In such areas that are involved in various RSNs, effective connectivity could be a useful technique in better understanding the role of the region or direction of information flow under different conditions. In accordance with functional connectivity studies, this method has also demonstrated the existence of modularity [26] and hierarchically organized information processing [27].

### Dynamic Relationships of RSNs

An important result of our analysis is the relative separation between networks that are strongly associated with one or the two large systems (eg., DMN and DAN) and networks that are more neutral. In both the seven and eleven cluster results, the LAN network and the VAN were least correlated with the task-positive and task-negative systems. Furthermore, the areas of uncertainty in the seven cluster result were similar to the LAN network, FPC network, and the VAN, and the areas of uncertainty in the eleven cluster result were most similar to FPC2.

In principle, our results can be related to the functional role that different networks play in cognition. It is well established that RSNs are related to structural connectivity but this relation is far from one-to-one. For example, regions of cortex like MT that have bidirectional anatomic connections to both visual and parietal cortex tend to cohere much more strongly with parietal than visual regions [28]. This indicates gating of anatomical connectivity by functional factors. Similarly, the strength of functional connectivity is not predictive of the presence of direct anatomical connections [29]. Finally, as demonstrated by recent MEG studies, RSNs have dynamic structures on time scales that are too rapid for anatomical connections [30], [31].

One interpretation of functional connectivity is that it reflects the history of task co-activation of specific sets of brain regions, i.e., essentially a Hebbian construct, as well as genetic factors [32]. This view is supported by developmental [33] and learning studies [34], [35]. According to the view that networks that statistically fire together will functionally wire together, networks that maintain a strong coherence in the resting state will also be coherent during common patterns of task activation. This is certainly true for the networks most strongly associated with the two main systems. The SMN, VIS network, and DAN tend to be positively activated during goal-driven tasks in the environment (e.g., sensorimotor, attention, decision tasks), while the DMN is strongly de-activated by the same tasks [36], [37]. More neutral networks such as the VAN tend to have variable relationships with other networks during task-activation studies. For instance, the VAN is independent from the DAN that is specifically recruited during the allocation of spatial attention; the VAN is also relatively suppressed, as compared to the DAN, during tasks that require perceptual filtering; finally, the VAN is co-activated with the DAN during target detection. Corbetta et al have argued that the VAN functions to reset or switch off other networks at the end of a cognitive task [38]. Hence it would be expected that the VAN is less consistently coherent with other networks at rest. A similar argument could be applied to other networks, such as the LAN or FPC networks, that may have “control” functions, hence dynamically change from moment-to-moment.

### Subcortical Involvement in RSNs

One significant difference in our analysis compared to past studies [10], [13], [39] is the inclusion of subcortical structures. Subcortical involvement was seen in many RSNs but was most prominent in the language and ventral attention networks.

### Language Network

The LAN network found using our method was slightly left lateralized and included regions beyond the traditional language areas. In our eleven network result, the LAN network was roughly divided into a lateral and medial cluster. The lateral language cluster consisted of bilateral cortical regions that incorporated Wernicke’s and Broca’s areas on the left. The medial cluster included the caudate, mesial frontal lobe, and inferior temporal lobe. While the lateral cluster would be fully expected given the long history of lesional and task-based imaging [40], [41], the medial cluster is also consistent with previous literature. Surgical or ischemic injuries of both the left lateral caudate and the medial frontal lobe have been associated with transient aphasias [42], [43]. Aphasia has also been linked to lesions in the basal ganglia [44]. Taken together, these findings suggest that the LAN network is indeed broadly distributed across a multitude of cortical and subcortical systems.

The LAN network was also related to the DMN. There was some spatial overlap between the LAN network and the DMN in temporal areas. This may be attributable to the ability of our clustering method to assign a voxel to more than one cluster. In the eleven cluster result, one of the language cluster centroids was also highly correlated to the DMN. In support of this finding, a task based fMRI study showed that areas of the DMN, including the anterior and posterior cingulate, precuneus, and medial frontal cortex, were modulated by auditory or audiovisual narratives in conjunction with superior temporal cortex [45].

### Ventral Attention

The cortical portion of the VAN network was very similar to that found in [13]. The VAN found by our method was slightly right lateralized and also had significant subcortical involvement. In support of this finding, in the parcellation by Doucet et al. [12], the module containing cortical structures belonging to the “salience” network also contained a RSN including basal ganglia. The eleven cluster result divided the VAN into two clusters, one of which was comprised mostly of subcortical structures. Strong subcortical connections have previously been seen in a description of the “salience” network [8] and a task based study of attentional networks [46].

### Subcortical Involvement in Other RSNs

The SMN also had thalamic involvement of the ventral lateral posterior nuclei and the anterior pulvinar nuclei. Connections between the ventral lateral posterior nuclei and motor and premotor areas have been shown [47], [48]. The anterior pulvinar nuclei have also been functionally linked to somatosensory areas [47], [48]. The FPC and VIS networks both had caudate involvement. In a study of cortical-subcortical connections, the caudate was found to be most strongly correlated with prefrontal cortex but also weakly correlated with parietal and occipital cortices [47].

### Optimal Numbers of Clusters

Seven [13], [39] and seventeen clusters [13] were also found to be optimal in other studies. Our seven cluster result was very similar to that of Yeo et al. [13] but differed in that one of our clusters corresponded to language areas. In contrast, Yeo et al. found a limbic system in their seven network parcellation. This difference may be attributable to the inclusion of subcortical structures in our analysis. The DMN was consistent with past studies done using a variety of methods [2], [5], [10], [13], [39], [49]. As seen in other work, our VIS network spanned much of occipital cortex [6], [10], [13]. The DAN found in our study had more frontal involvement but included similar areas as compared to past studies [7], [10], [13]. The FPC network found in the seven cluster result was also in agreement with past reports [10], [11], [13]. In the eleven cluster result, FPC2 had less visual resemblance to the FPC network of past studies. However, Figure 6 illustrates that the areas with the highest degree of shared membership between different clusters visually resemble areas belonging to the FPC network, particularly FPC2. The FPC2 clusters of the two study groups were spatially least similar in the eleven cluster result. Furthermore, although this cluster was named FPC2 because it was most similar to the FPC network from the seven cluster result, the inner product value was lowest among all the eleven clusters, thus indicating that it may not be a division of the FPC network. Further work is needed to determine the functional significance of this cluster.

We found that eleven clusters also minimized cluster dispersion. Although this number has not been described previously, the eleven cluster result related to the task-negative and task-positive systems with the same pattern of positive and negative correlations as the seven cluster result. In the eleven cluster result, the FPC network, LAN network, VAN, and SMN were divided into two components each. The functional significance of the division of the FPC network into two components requires further investigation. The LAN network and the VAN were each approximately divided into cortical and subcortical clusters.

The division of our SMN in the eleven cluster result contrasts with the dorsal-ventral division in the 17 network parcellation by Yeo et al. [13]. However, our SMN contained more of the insular cortex compared to the seven network parcellation by Yeo et al. [13]. The anterior-posterior division of insular cortex seen in our division of the SMN has also been seen in other resting state [50], [51] and diffusion studies [52]. The SMA and pre-SMA division has also been seen by others [52]–[54]. In our division of the SMN, the posterior insula was clustered with primary somatosensory areas and the SMA and the anterior insula was clustered with the pre-SMA. In resting state studies, the posterior insula was associated with sensorimotor cortex and the posterior cingulate [50] and the SMA was associated with primary motor cortex, somatosensory cortex, premotor areas, and the middle cingulate cortex [55]. Furthermore, the anterior insula was associated with middle and inferior temporal cortex and anterior cingulate was thought to be involved with limbic function [50], and the pre-SMA was associated with prefrontal cortex, inferior frontal gyrus, angular gyrus, and anterior cingulate cortex and thought to be involved with cognitive functions [55].

### Application of the Fuzzy-c-means Clustering Algorithm to Find RSNs

In the current study, we also demonstrated the usefulness of the fuzzy-c-means clustering algorithm in exploring resting state activity. In our analysis, the fuzzy-c-means algorithm was able to consistently find seven RSNs in the averaged data of 17 and 21 healthy individuals. Many runs of the algorithm with random initialization provided consistent results, and non-random initialization using *a priori* knowledge about the networks yielded identical results.

A variety of methods have been used to study resting state BOLD activity, including seed-based methods [3], [7], [11], independent components analysis [4]–[6], [12], [49], graph methods [10], and effective connectivity [26], [27]. The k-means clustering algorithm [15], and the expectation-maximization clustering algorithm [13] have also been used. Although several alternative methods exist for the analysis of resting state BOLD data, the fuzzy-c-means clustering algorithm contrasts with these methods in several ways. In comparison to seed-based methods, which require *a priori* selection of regions of interest, the fuzzy-c-means clustering algorithm is data driven and allows one to consider all of the data simultaneously without prior information. Graph methods are also applied on a set of predefined regions of interest. In contrast, independent components analysis is also data driven but requires judgment from the user concerning which components belong to RSNs and which are non-physiological, introducing a possible source of subjectivity. The fuzzy-c-means clustering algorithm is designed to find exactly the number of pre-set clusters. Although we have shown that the fuzzy-c-means algorithm is robust when analyzing a group average, further work needs to be done to evaluate its performance with noisier individual data. Most similar is the method used by Yeo et al. [13], but an apparent advantage of the fuzzy-c-means algorithm is that it is simpler to implement. Finally, an advantage that is particularly relevant to resting state data is that it allows for the weighted assignment of each voxel to more than one cluster. This greater flexibility may be more physiologically accurate as it is able to represent areas of shared functionality in the brain.

### Conclusion

In the present work, we demonstrate a hierarchical organization of resting state activity using the fuzzy-c-means clustering algorithm. We confirm the functional division of the brain into two large systems and further show how these systems further subdivide in hierarchical fashion. Our analysis also shows the importance of including subcortical structures, since they were involved in many RSNs and comprised significant components of the language and ventral attention networks. Finally, we demonstrated the reliability of the fuzzy-c-means clustering algorithm as a data driven approach for finding RSNs.

## Materials and Methods

### Ethics Statement

All participants gave written informed consent prior to taking part in the study as approved by the Washington University Institutional Review Board.

### Participants

Resting state BOLD fMRI data (3T, 4 mm isotropic voxels, TE 25 ms, TR 2.16 s) were obtained in two separate populations. Characteristics of the study populations are given in Table 2. The subjects were instructed to remain still and maintain fixation on a crosshair. All data were obtained for previous studies [56].

**Table 2:** Characteristics of the study populations.

	Group 1 (N = 17)	Group 2 (N = 21)
Number of men	8	7
Number right handed	17	21
Average age (range)	23.1 (18–27)	27.6 (23–35)
Total scan time	28 minutes	28 minutes

### Image Processing

The BOLD fMRI data were preprocessed according to previously published methods [56]. Preprocessing steps included compensation of systematic, slice-dependent time shifts, elimination of odd-even slice intensity differences due to interleaved acquisition, rigid body correction for head motion within and across runs, and signal intensity normalization to yield a whole brain mode value of 1000 (not counting the first four frames) [57]. Atlas registration was achieved by computing affine transforms connecting the first frame of the fMRI run (averaged over all runs after cross-run realignment) with the T2-weighted and T1-weighted structural images [57]. The fMRI data were transformed to an atlas representative template derived from 12 healthy individuals [58] and resampled to 3 mm cubic voxels. This step combined atlas transformation and movement correction within and across runs.

Linear trends across runs were removed voxelwise and the data were spatially smoothed with a 6 mm FWHM Gaussian kernel. The data were low-pass filtered to retain frequencies less than 0.1 Hz. Six movement parameters as well as their temporal derivatives were regressed out of the data on a voxelwise basis. The whole brain signal was regressed out from the data [56].

Following preprocessing, frames with excessive head motion were excluded using the DVARS method [59], [60]. Frames with DVARS greater than 0.5% were eliminated. On average, this method eliminated 3.3% of frames from study group 1 and 3.7% of frames from study group 2.

A mask including cortical and subcortical gray matter and excluding white matter and CSF was created using FreeSurfer (Boston, MA). Cortical reconstruction and volume segmentation were performed [61], and cortical and subcortical gray matter regions were selected. These areas were then masked with a thresholded image of average BOLD signal intensity to exclude areas prone to susceptibility artifacts.

### Clustering Algorithm

The fuzzy-c-means clustering algorithm [22] was implemented in Matlab (Natick, MA). Fuzzy-c-means is an iterative algorithm that assigns to each of *n* points to be clustered, *X_k_* for *k = 1,…,n,* a weighted membership between zero and one to each cluster. It requires *a priori* the number of clusters, *c*, and the initial location of the centroid, *V_i_* for *i = 1,…,c,* for each cluster. The starting locations can be chosen randomly or with prior knowledge about the data. The points and centroids are represented as vectors, with each element of the vector representing a dimension of information that can be used for clustering. For *n* points in the data, the membership of each point indexed by *k,* to each cluster indexed by *i,* is computed as:
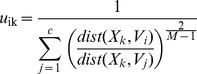
(1)where *u_ik_* is the membership of point *X_k_* to cluster *i*. Euclidean distance was used as the distance metric (*dist*). The parameter M is set at a value greater than 1 that optimizes the clustering [22]. A value of 1.2 was chosen for our data because it minimized cluster dispersion. With the new memberships of each point indexed by *k* to each cluster indexed by *i*, the locations of the centroids are updated as
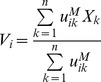
(2)for each centroid Vi. The weighted memberships and locations of the centroids are repeatedly updated until convergence is achieved. As a measure of convergence, we used the Xie-Beni Index, which is given as



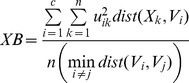
(3)
[62]. The algorithm was stopped when there was less than a 0.0001 change in the index over five consecutive iterations.

### Application of the Fuzzy-c-means Algorithm to Resting State Data

The fuzzy-c-means algorithm was applied to BOLD data averaged over all subjects in each of the two groups. For each subject, a gray matter mask was applied to extract the time course of 18,611 voxels in cortical gray matter, basal ganglia, and thalamus. The Pearson’s correlation was calculated between the timecourses of every pair of voxels, resulting in an 18,611 by 18,611 correlation map. The average correlation maps of subjects for each of the groups were then clustered using the fuzzy-c-means algorithm. In this formulation, each of the *n* = 18,611 voxels was represented by its correlation to all other voxels in 18,611-dimensional space. The number of clusters, *c* was set between 2 and 20.

The initial points of the centroids were chosen randomly and non-randomly. For random initialization, voxels within a randomly chosen cube of 3 voxels in each dimension were averaged as the starting location of each centroid. Since the initial locations of the centroids can affect the clustering result, all results with random initialization are given as the average of 20 runs of the algorithm. When chosen non-randomly, the voxels within a 5 mm radius sphere around regions of interest were averaged to provide the starting location of each centroid. Using Talairach coordinates [63], regions of interest were chosen based on past studies implicating their involvement in one of seven resting state networks: precuneus (0, −65, +31) for the DMN [2], right dorsolateral prefrontal cortex (+43, +22, +34) for the FPC [11], left superior temporal gyrus (−54, −23, −03) for the language network (LAN), right middle frontal gyrus (+24, +38, +25) for the VAN [7], left parietal operculum (−45, −30, +22) for the somatomotor network, superior occipital gyrus (−13, −93, +18) for the visual network, and right frontal eye field (+23, −8, +55) for the DAN [7], [11].

### Cluster Dispersion Measure

A cluster dispersion (*CD*) measure was used to evaluate the clustering results obtained using different numbers of clusters. This metric was calculated as.
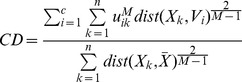
(4)where 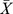 is the average over all gray matter voxels (the denominator is the equivalent of the numerator in the case that all voxels belong to a single cluster). In an optimal clustering result, members are closer to their centroids giving a smaller cluster dispersion measure.

### Spatial and Temporal Similarity for Cluster Classification

In order to objectively classify each of the clusters as one of the resting state networks, an inner product was computed between each cluster’s 18,611 length weight vector and a set of reference weight vectors. This is referred to as spatial similarity. For two weight vectors *u_1_* and *u_2_*, spatial similarity (*SS*) was calculated as.
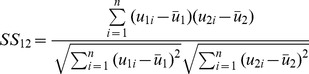
(5)


The seven reference weight vectors were those found using 20 runs of the algorithm with random initialization. The identities of the reference weight vectors were determined based on visual comparison with past studies. When new results were computed with a number of clusters other than seven, a best match assignment was made for each of the new clusters.

We primarily used spatial similarity in the analysis, since it best corresponds to the visual impression of similarity. However, when classifying a large number of clusters where each cluster included a much smaller number of voxels, temporal similarity was used to determine the identities of the clusters. Temporal similarity was measured using the inner product of the time courses, or centroids, of the compared clusters. For two centroids *V_1_* and *V_2_* with *d* elements indexed by *t*, temporal similarity (*TS*) was calculated as.
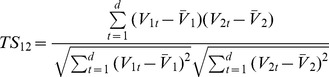
(6)


### Classification Uncertainty

To determine the uncertainty with which each voxel was classified, the geometric mean of each voxel’s weights was taken, where larger values indicated greater uncertainty and smaller values indicated a high degree of membership to one cluster. The classification uncertainty (*CU*) for a voxel *i* was calculated as:
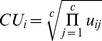
(7)


## Supporting Information

Figure S1 **The seventeen cluster result had two subdivisions of the A) DMN, D) VAN, F) VIS network, and G) DAN.** It had three subdivisions of the B) FPC network, C) LAN network, and E) SMN.
(TIF)

Table S1 **Spatial and temporal inner products between the two cluster result and the clusters between 3 and 7.**
(DOCX)

Table S2 **Spatial and temporal inner products between incremental changes in the cluster number from 2 to 7.**
(DOCX)
