# Supplementary figures and images for: Clustering of Resting State Networks
Source: PLoS One. 2012 Jul 9;7(7):e40370. doi: 10.1371/journal.pone.0040370 (PMC3392237; doi:10.1371/journal.pone.0040370)

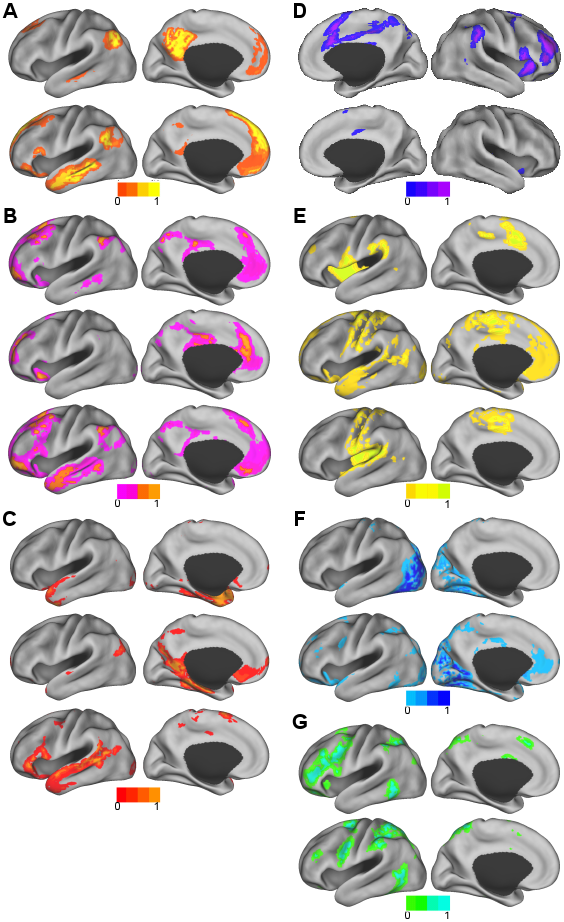

Supplement: Figure S1 — The seventeen cluster result had two subdivisions of the A) DMN, D) VAN, F) VIS network, and G) DAN. It had three subdivisions of the B) FPC network, C) LAN network, and E) SMN. (TIF) [file pone.0040370.s001.tif]
