# Supplementary material for: Clustering of Resting State Networks
Source: PLoS One. 2012 Jul 9;7(7):e40370. doi: 10.1371/journal.pone.0040370 (PMC3392237; doi:10.1371/journal.pone.0040370)
Supplement: Table S1 — Spatial and temporal inner products between the two cluster result and the clusters between 3 and 7. (DOCX) [file pone.0040370.s002.docx]

Table S1

| Spatial | | | | | | | |
| --- | --- | --- | --- | --- | --- | --- | --- |
|  | DMN3^c^ | TN3 | SMN3 |  |  |  |  |
| TN^a^ | 0.8925 | -0.2107 | -0.771 |  |  |  |  |
| TP^b^ | -0.8925 | 0.2107 | 0.771 |  |  |  |  |
|  |  |  |  |  |  |  |  |
|  | DMN4 | VAN4 | SMN4 | VIS4 |  |  |  |
| TN | 0.7982 | 0.1699 | -0.5791 | -0.4299 |  |  |  |
| TP | -0.7982 | -0.1699 | 0.5791 | 0.4299 |  |  |  |
|  |  |  |  |  |  |  |  |
|  | DMN5 | LAN5 | VAN5 | SMN5 | VIS5 |  |  |
| TN | 0.7193 | 0.1264 | 0.1397 | -0.5167 | -0.4882 |  |  |
| TP | -0.7193 | -0.1264 | -0.1397 | 0.5167 | 0.4882 |  |  |
|  |  |  |  |  |  |  |  |
|  | DMN6 | FPC6 | LAN6 | VAN6 | SMN6 | VIS6 |  |
| TN | 0.6863 | 0.1696 | 0.1387 | -0.0502 | -0.495 | -0.4634 |  |
| TP | -0.6863 | -0.1696 | -0.1387 | 0.0502 | 0.495 | 0.4634 |  |
|  |  |  |  |  |  |  |  |
|  | DMN | FPC | LAN | VAN | SMN | VIS | DAN |
| TN | 0.6058 | 0.5218 | 0.1445 | -0.1186 | -0.456 | -0.3651 | -0.3558 |
| TP | -0.6058 | -0.5218 | -0.1445 | 0.1186 | 0.456 | 0.3651 | 0.3558 |
|  | | | | | | | |
| Temporal | | | | | | | |
|  | DMN3 | TN3 | SMN3 |  |  |  |  |
| TN | 0.997 | -0.5802 | -0.879 |  |  |  |  |
| TP | -0.9312 | 0.5586 | 0.9875 |  |  |  |  |
|  |  |  |  |  |  |  |  |
|  | DMN4 | VAN4 | SMN4 | VIS4 |  |  |  |
| TN | 0.9899 | 0.3478 | -0.8404 | -0.7388 |  |  |  |
| TP | -0.9413 | -0.1136 | 0.9692 | 0.576 |  |  |  |
|  |  |  |  |  |  |  |  |
|  | DMN5 | LAN5 | VAN5 | SMN5 | VIS5 |  |  |
| TN | 0.9903 | 0.2755 | 0.2931 | -0.8037 | -0.8267 |  |  |
| TP | -0.9379 | -0.3113 | -0.0804 | 0.9465 | 0.7122 |  |  |
|  |  |  |  |  |  |  |  |
|  | DMN6 | FPC6 | LAN6 | VAN6 | SMN6 | VIS6 |  |
| TN | 0.9881 | 0.2357 | 0.2878 | 0.0352 | -0.8185 | -0.8022 |  |
| TP | -0.9376 | -0.1335 | -0.3353 | 0.2122 | 0.9516 | 0.6652 |  |
|  |  |  |  |  |  |  |  |
|  | DMN | FPC | LAN | VAN | SMN | VIS | DAN |
| TN | 0.9792 | 0.8067 | 0.2938 | -0.0514 | -0.8204 | -0.7108 | -0.6902 |
| TP | -0.935 | -0.7013 | -0.3435 | 0.2961 | 0.9479 | 0.5475 | 0.6959 |

^a^Task-negative

^b^Task-positive

^c^The names of the intermediary networks are formed from their closest match in the group of 2 or 7 with a number attached to denote the number of clusters in that grouping.
