# Supplementary material for: Clustering of Resting State Networks
Source: PLoS One. 2012 Jul 9;7(7):e40370. doi: 10.1371/journal.pone.0040370 (PMC3392237; doi:10.1371/journal.pone.0040370)
Supplement: Table S2 — Spatial and temporal inner products between incremental changes in the cluster number from 2 to 7. (DOCX) [file pone.0040370.s003.docx]

Table S2

| Spatial | | | | | | | |
| --- | --- | --- | --- | --- | --- | --- | --- |
|  | DMN3^c^ | TN3 | SMN3 |  |  |  |  |
| TN^a^ | 0.8925 | -0.2107 | -0.771 |  |  |  |  |
| TP^b^ | -0.8925 | 0.2107 | 0.771 |  |  |  |  |
|  |  |  |  |  |  |  |  |
|  | DMN4 | VAN4 | SMN4 | VIS4 |  |  |  |
| DMN3 | 0.9506 | -0.0966 | -0.4543 | -0.476 |  |  |  |
| TN3 | -0.5211 | 0.4362 | -0.4737 | 0.6486 |  |  |  |
| SMN3 | -0.5534 | -0.2896 | 0.9167 | -0.0727 |  |  |  |
|  |  |  |  |  |  |  |  |
|  | DMN5 | LAN5 | VAN5 | SMN5 | VIS5 |  |  |
| DMN4 | 0.9643 | -0.1185 | -0.2236 | -0.351 | -0.3514 |  |  |
| VAN4 | -0.3092 | 0.1509 | 0.8852 | -0.3011 | -0.323 |  |  |
| SMN4 | -0.3525 | -0.3277 | -0.2514 | 0.9542 | -0.0661 |  |  |
| VIS4 | -0.3978 | 0.3259 | -0.3222 | -0.3207 | 0.751 |  |  |
|  |  |  |  |  |  |  |  |
|  | DMN6 | FPC6 | LAN6 | VAN6 | SMN6 | VIS6 |  |
| DMN5 | 0.9865 | -0.0882 | -0.2159 | -0.2735 | -0.2983 | -0.2974 |  |
| LAN5 | -0.2339 | -0.2289 | 0.9896 | -0.1449 | -0.2872 | -0.1739 |  |
| VAN5 | -0.2432 | 0.6273 | -0.2151 | 0.7858 | -0.2598 | -0.2689 |  |
| SMN5 | -0.2981 | -0.2785 | -0.2831 | -0.0759 | 0.9814 | -0.2076 |  |
| VIS5 | -0.3015 | 0.0199 | -0.2208 | -0.2199 | -0.1837 | 0.9545 |  |
|  |  |  |  |  |  |  |  |
|  | DMN | FPC | LAN | VAN | SMN | VIS | DAN |
| DMN6 | 0.958 | 0.126 | -0.2018 | -0.2314 | -0.2608 | -0.2286 | -0.2323 |
| FPC6 | -0.246 | 0.7114 | -0.2404 | -0.0545 | -0.2874 | -0.2238 | 0.4696 |
| LAN6 | -0.1858 | -0.1624 | 0.994 | -0.13 | -0.2512 | -0.1009 | -0.211 |
| VAN6 | -0.2586 | 0.0875 | -0.2082 | 0.9397 | -0.2097 | -0.2282 | -0.0012 |
| SMN6 | -0.2503 | -0.2625 | -0.2589 | -0.0717 | 0.9828 | -0.1783 | -0.0784 |
| VIS6 | -0.2505 | -0.2515 | -0.1892 | -0.2252 | -0.173 | 0.9162 | 0.2501 |
|  | | | | | | | |
| Temporal | | | | | | | |
|  | DMN3 | TN3 | SMN3 |  |  |  |  |
| TN | 0.997 | -0.5802 | -0.879 |  |  |  |  |
| TP | -0.9312 | 0.5586 | 0.9875 |  |  |  |  |
|  |  |  |  |  |  |  |  |
|  | DMN4 | VAN4 | SMN4 | VIS4 |  |  |  |
| DMN3 | 0.995 | 0.3078 | -0.8477 | -0.7216 |  |  |  |
| TN3 | -0.5962 | -0.2214 | 0.4261 | 0.6592 |  |  |  |
| SMN3 | -0.9026 | -0.0117 | 0.9927 | 0.4533 |  |  |  |
|  |  |  |  |  |  |  |  |
|  | DMN5 | LAN5 | VAN5 | SMN5 | VIS5 |  |  |
| DMN4 | 0.9993 | 0.2927 | 0.1737 | -0.8359 | -0.7676 |  |  |
| VAN4 | 0.2339 | -0.2184 | 0.9544 | 0.0636 | -0.6118 |  |  |
| SMN4 | -0.8627 | -0.322 | 0.048 | 0.9939 | 0.5399 |  |  |
| VIS4 | -0.6745 | 0.0525 | -0.7425 | 0.3342 | 0.9139 |  |  |
|  |  |  |  |  |  |  |  |
|  | DMN6 | FPC6 | LAN6 | VAN6 | SMN6 | VIS6 |  |
| DMN5 | 0.9993 | 0.2039 | 0.2784 | -0.08 | -0.8399 | -0.7354 |  |
| LAN5 | 0.2856 | -0.6815 | 0.9975 | -0.2681 | -0.2508 | -0.2248 |  |
| VAN5 | 0.17 | 0.7472 | -0.5105 | 0.8726 | 0.007 | -0.59 |  |
| SMN5 | -0.8301 | -0.1675 | -0.2875 | 0.3921 | 0.9983 | 0.4246 |  |
| VIS5 | -0.7647 | -0.1136 | -0.3272 | -0.4015 | 0.4962 | 0.9807 |  |
|  |  |  |  |  |  |  |  |
|  | DMN | FPC | LAN | VAN | SMN | VIS | DAN |
| DMN6 | 0.9973 | 0.7545 | 0.3133 | -0.176 | -0.8326 | -0.6261 | -0.7041 |
| FPC6 | 0.0992 | 0.7411 | -0.6758 | 0.1861 | -0.2317 | -0.4558 | 0.4834 |
| LAN6 | 0.3593 | -0.2578 | 0.9993 | -0.1743 | -0.2545 | -0.0428 | -0.6734 |
| VAN6 | -0.1334 | 0.3064 | -0.3354 | 0.9728 | 0.3022 | -0.562 | 0.0904 |
| SMN6 | -0.8321 | -0.6436 | -0.2874 | 0.4411 | 0.9986 | 0.3608 | 0.5224 |
| VIS6 | -0.7079 | -0.7306 | -0.2152 | -0.423 | 0.4752 | 0.9636 | 0.5977 |

^a^Task-negative

^b^Task-positive

^c^The names of the intermediary networks are formed from their closest match in the group of 2 or 7 with a number attached to denote the number of clusters in that grouping.
